# Delirium prevalence and incidence in acutely admitted older patients: an observational cohort study

**DOI:** 10.1186/s12877-025-06903-8

**Published:** 2025-12-19

**Authors:** Mathias Nikolai Petersen Hella, Hogne Soennesyn, Hanne Brit Hetland, Audun Osland Vik-Mo, Ane Djuv, Anita Sunde, Rune Tord Wathne Oftedal, Leiv Otto Watne, Dag Aarsland, Anne Katrine Bergland

**Affiliations:** 1 Centre for Age-Related Medicine, Stavanger University Hospital, Stavanger, Norway; 2 Department of Clinical Medicine, University of Bergen, Bergen, Norway; 3 Department of Research, section of biostatistics, Stavanger University Hospital, Stavanger, Norway; 4 Department of Orthopaedic Surgery, Stavanger University Hospital, Stavanger, Norway; 5 Oslo Delirium Research Group, Institute of Clinical Medicine, University of Oslo, Oslo, Norway; 6 Department of Geriatric Medicine, Akershus University Hospital, Lørenskog, Norway; 7 Department of Old Age Psychiatry, Institute of Psychiatry, Psychology and Neuroscience, King’s College London, London, UK

**Keywords:** Delirium, Prevalence, Occurrence, Older adults, Incidence, Acute-hospital, Emergency-department, Outcomes, Mortality, Length-of-stay, Screening, 4AT, Under-reported

## Abstract

**Background:**

Delirium is common in acutely ill older adults and is associated with multiple unfavourable outcomes, including an increased risk of dementia and death. As the population ages and more people live with dementia, updated and accurate estimates of delirium prevalence are important. The primary aim of this study was to identify delirium point prevalence in the emergency department and incidence during hospitalisation. Our secondary aim was to compare outcomes: length of stay, need for a higher level of care, and mortality after discharge in patients with and without delirium.

**Methods:**

In this unselected observational cohort study, all older adults aged 65 years or above acutely admitted to the emergency department of a large Norwegian hospital during a 5-day and 4-night midweek period, were screened for delirium by the 4 “A”s test. A final consensus delirium diagnosis was made based on review of all available information in the patients’ electronic health record to consider if The Diagnostic and Statistical Manual of Mental Disorders, Fifth Edition’s criteria (DSM-V) for delirium were fulfilled.

**Results:**

Of 240 patients assessed, 14% (*n* = 33) fulfilled DSM-V criteria for delirium in the emergency department, and 8% (*n* = 17) of the remaining 207 patients developed delirium later during the hospitalisation. Only 4 of the 50 patients with delirium (8%) had a documented diagnosis of delirium in their discharge summary. For patients with delirium, the current hospital admission was more often a readmission (42% vs. 18%, *p* < 0.001). Delirium patients also had longer hospitalisations (4 vs. 2 days, *p* < 0.001), and higher 9-month mortality (52% vs. 14%, *p* = 0.002), corrected for age, sex and severity of acute illness by The National Early Warning Score 2.

**Conclusions:**

Delirium was common and underdiagnosed in our study and associated with unfavourable outcomes for the patients. Delirium prevention, identification and management should be key priorities for the health care system.

**Trial registration:**

Our article does not report results of a health care intervention, and the protocol is not registered in a trial registry.

**Supplementary Information:**

The online version contains supplementary material available at 10.1186/s12877-025-06903-8.

## Strengths and limitations of this study

- We included 92% of all acutely admitted patients in the study period, which is high compared to similar studies and a strength in providing a precise and updated estimate of delirium occurrence.
- We registered both delirium prevalence in the emergency department and incidence during the rest of the hospitalisation.
- Delirium assessment was done systematically and independently by two geriatricians, with high interrater reliability, based on The Diagnostic and Statistical Manual of Mental Disorders, Fifth Edition’s criteria.
- We compared outcomes for patients with and without delirium, but without data on comorbidities, which is a limitation.

## Background

Delirium is an acute condition affecting cognition, arousal and attention, with underlying causes that should be identified and treated properly [1, 2]. Older patients often present with delirium when acutely ill, and old age, dementia, surgery and frailty are well known risk factors for delirium [2, 3].

Delirium prevalence and incidence varies in different patient populations [2]. In intensive care units up to 70% of patients develop delirium [2]. A 2020 meta-analysis found that approximately 25% of older hospital admitted patients develop delirium [4]. This study did however only include studies on medical and geriatric inpatients, which may constrain the applicability of the estimates of occurrence to other settings, such as that of all acutely admitted patients. Delirium is often underdiagnosed [5], and is associated with negative outcomes, including longer hospitalisations, institutionalisation, and increased mortality [6, 7]. Several studies show an association between delirium and an increased risk of cognitive decline and dementia [6, 8, 9]. Delirium also increases the cost of hospitalisation and follow-up after discharge [10]. Multicomponent interventions can prevent delirium [11, 12] and are likely cost-effective [13]. International guidelines for good management of delirium in different settings include advice on treating the causes of delirium and optimising multiple factors associated with sustained delirium [14, 15]. Few guidelines exist specifically for the emergency department (ED) setting, but screening to identify high-risk patients and the use of multicomponent interventions for risk reduction and management are recommended in an umbrella review [16].

Patient admissions in the emergency departments are increasing yearly, with the largest increase in the age group 67–79 years [17]. The number of people living with dementia is also increasing [18, 19]. As dementia and old age are important risk factors for delirium development [2], and life expectancy years and number of people living with dementia increase in high income countries, this may affect the prevalence of delirium in hospitalised older adults. Delirium prevention, identification and treatment are important for future sustainability of the health care system, optimising use of resources and reducing costs [13]. Updated estimates of delirium prevalence will likely have consequences for clinical practice, as well as implications for the distribution of healthcare resources to best treat the increasing number of ageing patients.

The primary objective of this study was to estimate the delirium occurrence in an unselected cohort of acutely admitted older adults, both in the emergency department (point prevalence) and during the consecutive hospitalisation (incidence). Our secondary objective was to compare outcomes: length of stay, need for higher level of care after discharge and 9-month mortality, for patients with and without delirium.

## Methods

### Study design

Observational unselected cohort study.

### Study setting

Stavanger University Hospital (SUH) is a large Norwegian hospital with an emergency department (ED) with approximately 100–120 daily admissions. SUH serves a population of approximately 390 000 patients and is the sole hospital in its catchment area. About 50% of the acutely admitted patients are 65 years or above.

### Study participants, inclusion and data registered

We included all patients, aged 65 years or above, acutely admitted to the emergency department (ED) at Stavanger University Hospital, for 5 days, including 4 nights. The screening period lasted from Monday, 02.10.2023, 08:00 a.m. until Friday, 06.10.2023, 08:00 p.m. For patients admitted more than once during this period, only the last admission was included. Patients admitted with life-threatening conditions and those who were expected to die during or shortly after admission were also included. The delirium screening results were documented in the patient’s electronic charts. Patients not speaking Norwegian or English were excluded unless an interpreter was available. For included patients, we analysed data on sex, age and whether the admission represented a readmission. We used the Norwegian Directorate of Health’s definition of readmission: an acute admission between 8 h and 30 days after the last hospital discharge [20]. We also registered which department the patients were admitted to, if they were discharged directly home from the ED and whether they had a known diagnosis of dementia. The National Early Warning Score 2 (NEWS2), a system for identifying clinical deterioration in patients in the ED [21], was registered at admission, as well as length of hospital stay (in whole days), and if delirium was documented as a formal diagnosis in the discharge summary. We also registered whether the admitted patients needed a higher level of care, such as home nursing care or temporary non-hospital institutional stays, at discharge compared to prior to admission. Mortality until 9 months after discharge was also registered.

### Delirium screening and diagnosis

Initial delirium screening in the emergency department was done as soon as possible by the 4 “A”s test (4AT)- a rapid screening test for delirium, with high sensitivity and specificity [22]. If the 4AT score was ≥ 4, the study personnel performed the Richmond Agitation Sedation Scale (RASS) [23] and the Observational Scale of Level of Arousal (OSLA) [24] to evaluate the patient’s level of agitation and arousal. The study personnel, all trained in performing the 4AT, RASS and OSLA, assisted the staff in the ED in performing the screenings. When delirium was suspected, the responsible nurse or doctor in the ED was informed. The delirium screening results were documented in the patient’s electronic charts. No additional delirium screening, as part of this study, was performed for the rest of the hospitalisation at the wards. Final delirium diagnoses, both for the point prevalence and incidence were done by retrospective chart review of all the available information in the patients’ health record, based on The Diagnostic and Statistical Manual of Mental Disorders, Fifth Edition’s (DSM-V) criteria for delirium [1]. Two geriatricians (MNPH and AKB) independently and retrospectively evaluated all available information from the patients charts from the hospital admission and stay to determine if the patients fulfilled a diagnosis of delirium based on DSM-V criteria. This evaluation was based on a method described previously [25], slightly modified to suit our study (Appendix 1). Patients with acute changes in cognition and disturbances in attention or awareness in the emergency department or later during hospitalisation, but not fulfilling all the DSM-V criteria, were classified as having subsyndromal delirium. Disagreements were solved through discussion with an old-age psychiatrist (AOVM). The diagnosis of delirium, by DSM-V criteria, showed high interrater reliability with a kappa of 0.92 (0.87–0.98).

### Statistical methods

The variables are reported as median (IQR) and number (percent). Normality was considered by the Shapiro-Wilk and Kolmogorov-Smirnov tests. Nonparametric tests were used for comparing delirium and no-delirium patients with not normally distributed data. Continuous variables were analysed using Mann-Whitney U test and categorical variables were analysed using Chi square (X^2^) statistics. Regression analyses used to compare outcomes for patients with and without delirium and adjusted for age, sex and NEWS2-score were performed in R version 4.3.2. Poisson regression was used to explore the length of stay, logistic regression to estimate the odds ratio for being discharged to a higher level of care, and Cox regression to test 9-month mortality. Survival curves (Kaplan Meyer plots) and figures were created using R version 4.3.2. The flowchart was created using Biorender. Interrater reliability kappa score was calculated in R-package vcd. A two-tailed p-value of < 0.05 was considered statistically significant and we used IBM SPSS Statistics version 26 software for the statistical analyses.

### Ethical considerations

We conducted the study in accordance with the World Medical Association Declaration of Helsinki and the Regional Committee for Medical and Health Research Ethics of Western Norway approved the study prior to its start, in line with the Norwegian Health Research act. This committee is appointed by the Norwegian Ministry of Education and Health. The participants did not need to actively consent but received written information with a possibility for reservation from participation in the study after discharge, as approved by the Regional Committee for Medical and Health Research Ethics (Western Norway; REK 424462). Patients who fulfilled inclusion criteria and died during or shortly after the study period were directly included as approved by the Regional Ethics Committee.

### Patient and public involvement

The project has been presented for a panel of user representatives at SESAM- Centre for Age-Related Medicine in Stavanger, and a dedicated user representative has been involved and consulted during the planning of this study.

## Results

### Patient enrolment

A total of 259 patients aged 65 years or above were admitted during the study period, 2.3% (*n* = 6) were excluded and 3.9% (*n* = 10) declined participation. We also excluded 1.2% (*n* = 3) of the patients presenting with critical illness where a conclusion regarding delirium was impossible to reach due to the patient’s clinical presentation in the emergency department.

**Fig. 1:**
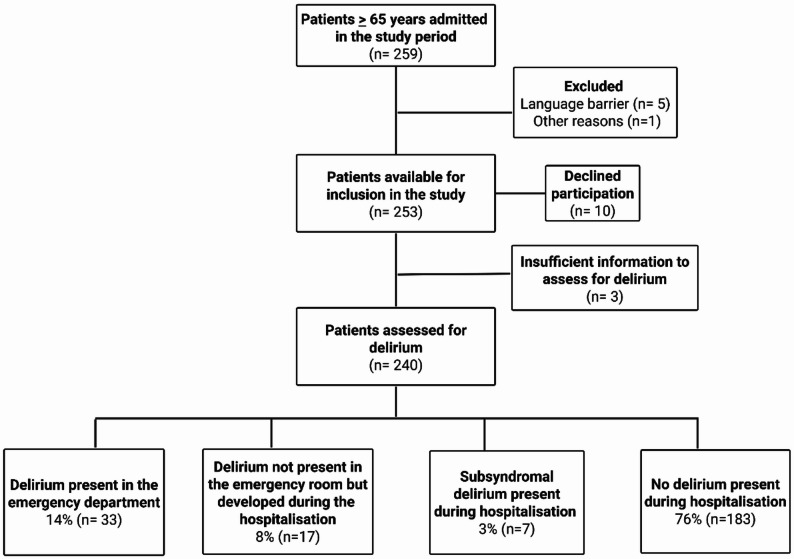
Flow chart presenting an overview of enrolment of acutely admitted patients to the emergency department in the study period, and the conclusion regarding delirium diagnosis according to The Diagnostic and Statistical Manual of Mental Disorders, Fifth Edition´s criteria

92.7% (*n* = 240) of all the admitted patients were eligible for inclusion and further analysis. Patient enrolment is shown in detail in Fig. 1.

### Delirium prevalence and incidence

Delirium point prevalence was 14% in the emergency department (ED), as 33 of 240 patients fulfilled DSM-V criteria for delirium. 17 of the 207 patients that did not have delirium in the ED developed delirium during the remaining hospital stay. The incidence of delirium development later during the hospitalisation was thus 8% (*n* = 17), and 3% (*n* = 7) fulfilled the criteria for a subsyndromal delirium. The total occurrence of delirium, both prevalent and incident, during the hospitalisation was 21% (50 of 240 patients).

### Study population characteristics

Population characteristics are shown in Table 1. The median age of the patients with delirium was significantly higher than that of the patients without delirium (82 (IQR 76–88) vs. 76 (70–83) years, *p* < 0.001). There was no significant sex difference between patients with and without delirium (54% (*n* = 27), vs. 41% (*n* = 75), *p* = 0.1). 94% (*n* = 47) of patients with delirium were 70 years of age or older. Median NEWS-score in the ED was higher for patients with delirium compared to patients without delirium (3 (IQR 0.5–5.5) vs. 1 (0–3), *p* = 0.004). A higher percentage of patients with delirium had a known diagnosis of dementia, compared to patients without delirium (14% (*n* = 7), vs. 1.6% (*n* = 3), *p* = 0.001). This hospital admission was a readmission for 23% (*n* = 54) of all the patients. Readmissions were more prevalent in patients with delirium compared to patients with no delirium (42% (*n* = 21) vs. 18% (*n* = 33), *p* < 0.001, see Table 1). Overall, patients were mostly admitted to the department of internal medicine 33% (*n* = 79), orthopaedic surgery 7% (*n* = 17) and surgical departments 13% (*n* = 31). Delirium patients showed a similar distribution regarding which departments they were admitted to.

**Table 1:** Population characteristics and comparisons for acutely admitted patients > 65 years and with and without delirium by DSM-V criteria during the hospitalisation

Characteristic	Overall *N* = 233^*1*^	Delirium anytime during hospitalization *N* = 50^*1*^	No delirium during hospitalization *N* = 183^*1*^	*p*-value^2^
Age	77 (72, 84)	82 (76, 88)	76 (70, 83)	< 0.001
Sex				0.10
Male	131(56%)	23(46%)	108(59%)	
Female	102(44%)	27(54%)	75(41%)	
NEWS score ED	2.00 (0.00,3.00)	3.00 (0.50, 5.50)	1.00 (0.00, 3.00)	0.004
Unknown	47 (20)	7(14)	40(22)	
Known dementia*				0.001
No	223(96%)	43(86%)	180(98%)	
Yes	10(4.3%)	7(14%)	3(1.6%)	
Is the admission a readmission?				< 0.001
No	179(77%)	29(58%)	150(82%)	
Yes	54(23%)	21(42%)	33(18%)	
Delirium diagnosis at discharge?				0.002
No	229(98%)	46(92%)	183(100%)	
Yes	4(1.7%)	4(8.0%)	0(0%)	
Discharged home from ED?				< 0.001
No	174(75%)	48(96%)	126(69%)	
Yes	59(25%)	2(4.0%)	57(31%)	

^1^Median (IQR); n(%)

^2^Mann-Whitney u test; Pearson’s Chi-squared test; Fisher’s exact test

^*^Dementia was based on chart review from all the information in the patient’s hospital records related to the hospital admission in the study period to see if they had a documented diagnosis of dementia

### Delirium screening and diagnosis at discharge

Of all patients eligible for inclusion 96.7% (232 of 240) were screened by 4AT in the ED, and 70% (23 of 33) of the patients with delirium had a 4AT-score > 4, compared to 1% (2 of 207) of the patients with no delirium in the ED (*p* < 0.001). Among the patients fulfilling the criteria for a subsyndromal delirium during hospitalisation only 28.6% (2 of 7) had a 4AT score > 4 in the ED. For some of the acutely admitted patients (*n* = 8) we could not perform a delirium screening with the 4AT as they presented clinically unstable and were transferred to special units, such as the intensive care unit shortly after arriving in the ED. A total of 8% (*n* = 4) of patients with delirium by DSM-V criteria, both in the ED and during the consecutive hospitalisation, had a documented diagnosis of delirium at discharge from the hospital.

### Outcomes

A comparison of outcomes for patients with and without delirium is visualised in Table 2. We adjusted the outcomes for age, sex and the severity of acute illness by NEWS-2 score. Patients with delirium were more often discharged to a higher level of care, compared to patients without delirium, but the difference was not statistically significant (48% (*n* = 21) vs. 20% (*n* = 37), *p* = 0.156). The length of hospital stay in whole days was significantly longer in patients with delirium compared to patients without delirium (4 (IQR 2-9.5) vs. 2 (0–4), *p* < 0.001).

**Table 2:** Outcomes comparing acutely admitted patients > 65 years, with and without delirium during their hospitalisation: higher level of care, length of hospital stay and cumulative 9-month mortality, adjusted for age, sex and severity of acute illness by the National early warning score

Overall, *N* = 233^*1*^	Delirium anytime during hospitalisation, *N* = 50	No delirium during hospitalisation, *N* = 183			Estimates (95% CI)	*p*-value^1^
Higher level of care after hospitalisation, n (%) *	21 (42)	37 (20)	OR		1.83 (0.79; 4.22)	0.156
Length of hospital stay, median (IQR) *	4.0 (2.0-9.5)	2.0 (0.0–4.0)	OR		1.72 (1.48; 2.0)	< 0.001
Cumulative 9-month mortality, n (%) *	26 (52)	25 (14)	HR		2.90 (1.50; 5.62)	0.002

^1^Logistic regression for higher level of care

Poisson regression for length of hospital stay

Cox regression for cumulative mortality

^*^NEWS score is missing for n=50 patients

**Fig. 2:**
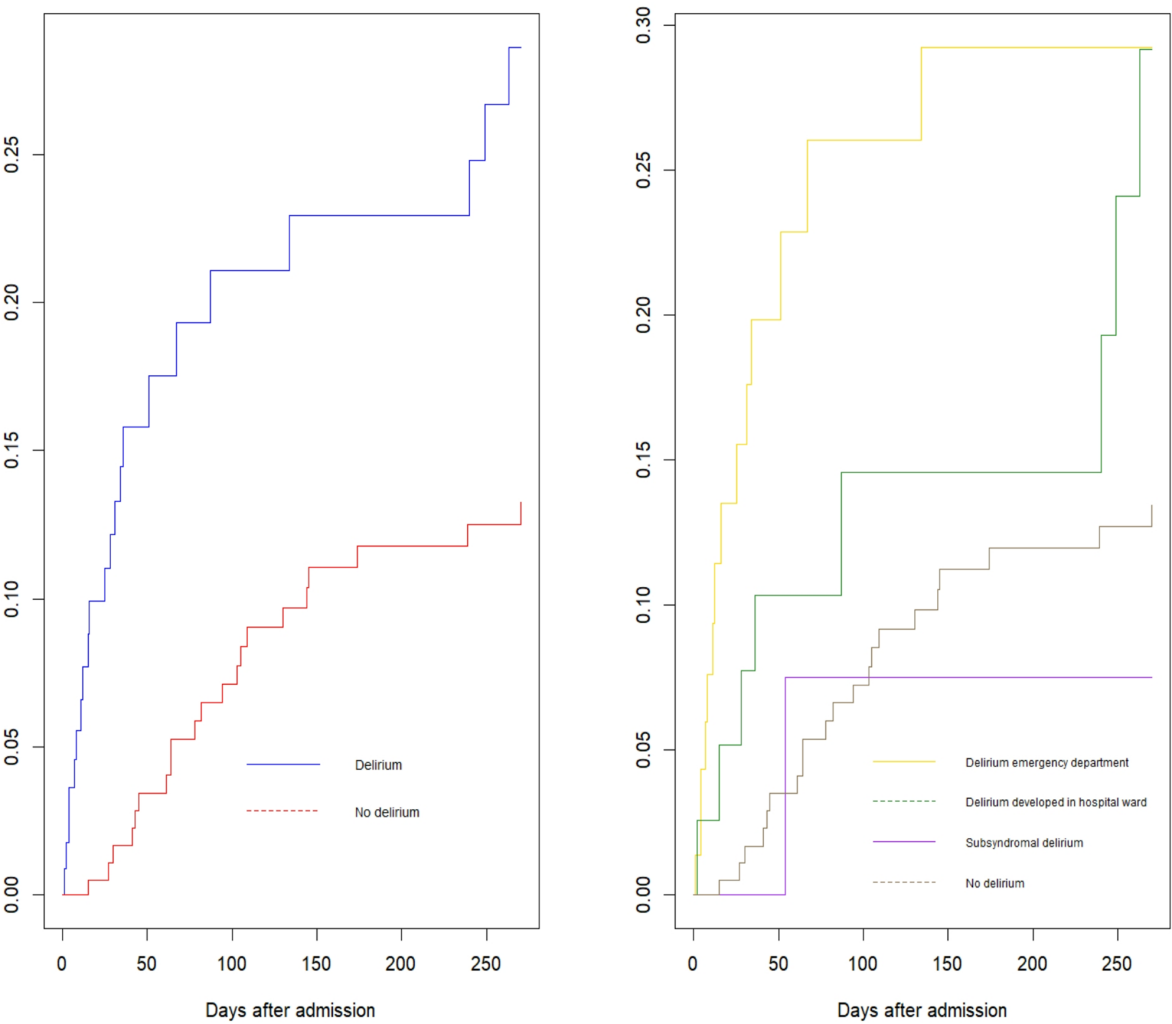
Kaplan-Meier plots, visualising mortality in acutely admitted patients aged > 65 years, with and without delirium during the hospitalisation, corrected for age, sex and NEWS2-score, showing a significant difference between the groups (left). The same patients divided into delirium subgroups; delirium present in the emergency department, delirium developed later during hospitalization, subsyndromal delirium during hospitalization and no delirium during hospitalization showing that the difference is mainly due to the patients with delirium prevalent in the emergency department (right)

For all patients included in this study, the 9-month mortality was 21%. Cumulative 9-month mortality was significantly higher for patients with delirium, compared to patients without delirium (52% (*n* = 26) vs. 14% (*n* = 25), *p* = 0.002), adjusted for age, gender and NEWS-2 score. Figure 2 shows Kaplan-Meier plots, comparing patients with and without delirium during the hospitalisation, corrected for age, sex and NEWS2-score. This figure shows a significant difference in cumulative 9-month mortality between patients with and without delirium.

## Discussion

This study found delirium to be common in acutely admitted older adults, with a point prevalence of 14% in the emergency department. The incidence was 8% during the rest of the hospitalisation for patients without delirium in the ED, giving a total occurrence of 21%. This is in line with estimates from a previous meta-analysis of published estimates of delirium occurrence in medical and geriatric inpatients over decades, showing an overall occurrence of 23% [4].

Our study design allowed for an unselected inclusion of all acutely admitted patients, which is important as many previous studies have examined specific cohorts of patients, such as medical geriatric patients [26], oncological patients [27], patients receiving non-invasive positive pressure ventilation [28], acute medical patients [29], or intensive care patients [30]. In later years the “delirium day” studies have aimed to estimate delirium point prevalence of acutely admitted patients, but with varying percentages of all acutely admitted patients included- such as 56% in a Norwegian study of medical and surgical wards [31] and 84% in a study across 108 acute and 12 rehabilitation wards in Italy [32]. It is a potential challenge in delirium studies that systematic assessments of capacity for consent, may lead to the exclusion of patients with delirium [33]. We included a very high percentage (92.7%) of all the acutely admitted patients in the study period without obtaining consent but instead gave the patients an opportunity for reservation after discharge, as approved by the ethical committee. A large international study conducted by the World Delirium Awareness Day team reported on delirium prevalence at two specific time points during one day in 2023 [34]. In this study, 2502 wards or units started, yet only 66.5% (*n* = 1664 wards) were analysed, meaning that even though the final number of patients was high the estimates of occurrence may have been biased. Ideally, delirium prevalence studies should include large numbers of patients and in addition include as many of the eligible patients as possible.

Many hospitals have implemented delirium screening in the emergency department, such as in the UK [35], yet this differs from our method of combining 4AT, RASS and OSLA and then retrospectively consider if the patients of the emergency department fulfil the criteria for delirium. Our method provides a robust tool for delirium diagnosis and combined with the high inclusion rate of all eligible patients gives an accurate estimate of particularly delirium point prevalence. This estimate can be transferable to other high-income countries with similar age-distribution. Stavanger University Hospital is also the sole hospital in its catchment area meaning that older patients with delirium were not admitted elsewhere, as could be the case in a setting where several hospitals share one catchment area.

The cut off for inclusion was ≥ 65 years in our study, and the median age of the patients included was 77 years. The patients with delirium were with a median age of 82 years significantly older than the non-delirious patients. Knowing that delirium risk is increased with higher age, the estimates would be expected to increase if we set the inclusion age higher, e.g. at > 75 years. Our estimated point prevalence is lower than reported in other studies [32] which could be both due to differences in age distribution of the patients but also due to differences in the diagnostic criteria applied to identify delirium. We chose 65 years as this is a commonly used definition of older adults, used among others by the United States National Institute of Aging [36]. Our finding that 94% of the patients with delirium were > 70 years is relevant in terms of which patient groups should be the focus of delirium identification. The National Institute for Health and Care Excellence guidelines highlight that patients aged > 65 years are at an increased risk of delirium development [15]. Yet, delirium identification in all admitted patients in this age group is resource demanding and a specification of which patients to prioritise for screening could be useful. Interestingly, for patients with delirium the current admission was a readmission for 42% compared to 18% for patients without a delirium. Potential reasons could be a higher burden of comorbidity or frailty in delirium patients. This finding is relevant information for health care personnel working in the emergency departments, particularly if resources are scant and not sufficient to perform delirium identification in all patients > 65 years.

The high screening percentage of 96.7% in our study period is not indicative of the general screening rate of delirium in our emergency department. In our study only 70% of the patients who fulfilled a delirium diagnosis by DSM-V criteria had a 4AT score of 4 or above, which is low considering the diagnostic accuracy of the 4AT described previously [22]. Our study has an unselected design meaning that 4AT could be less sensitive in this setting, which is supported by the findings of a sensitivity of 4AT of 76% in a randomised study performed in a similar setting [37]. The sensitivity of the 4AT in our study could also be due to local variability in performing the test or represent challenges in identifying delirium in certain patient groups. For patients with dementia, particularly Lewy body dementia, delirium screening and identification can be difficult due to overlapping symptoms [38, 39]. Delirium superimposed on dementia (DSD) represents a challenge and Fong et al. recommend modifying delirium screening instruments for this group particularly but also highlights that this would require validation [39]. Challenges in detecting delirium in certain patient groups could support repeated delirium screening in patients at risk of delirium with low scores on the initial screening. This is particularly relevant for patients with critical illness, hip fracture and dementia. If these patients present with symptoms of delirium and low scores on the screening test they may present with a subsyndromal delirium (SSD), meaning they fulfil some, but not all the criteria for a delirium [40]. 3% of the patients in our study presented with SSD during the hospitalisation. We did not find a higher 9-month mortality in this group, which could be explained by a low number of patients. SSD is nonetheless relevant as several previous studies have shown that patients with SSD have an increased risk of negative outcomes [40, 41]. In our study only 28% of patients with SSD during the hospitalisation had a score > 4 on the 4AT in the ED. This is important information for clinicians working with patients at risk for delirium. We found that delirium patients are admitted to most of the departments examined in our study, highlighting the need for delirium knowledge and awareness throughout the hospital. As demonstrated in our study, hospitals implementing delirium screening should validate the screening tool against established criteria for delirium diagnosis to determine to which extent patients with delirium are detected by the screening. Our hospital now has implemented delirium screening for older patients in the emergency department. Next steps include establishing other checkpoints for delirium screening at the wards and postoperatively in addition to following the delirium occurrence when we implement toolkits to prevent delirium in our patients. We should also evaluate the somewhat low 4AT sensitivity in our study compared to the DSM-V criteria to increase the future delirium detection by 4AT screening. These steps are generalisable for other hospitals aiming to prevent and detect delirium.

Only 8% of patients with delirium had a formal diagnosis documented in their electronic chart at discharge, which is low, but expected given results from other studies [5, 42]. This number stands in contrast to the identified delirium occurrence in our hospital and could indicate that most cases of delirium are never identified and thus not treated according to recommendations for good practice [14, 15]. Yet, it is uplifting to see that only 2 (4%) of patients with delirium were discharged directly home from the ED, indicating that the health care personnel have been aware of these patients and treated them in hospital. The increased risk of delirium development in dementia is well established [2, 43], and the patients with dementia had, as expected, a significantly higher occurrence of delirium in our study. The National Early Warning Score 2 (NEWS2) was significantly higher for delirium patients in our study, most likely indicating increased severity of the presenting illness in delirious patients. We found, in line with previous studies [6, 7], that delirium was significantly associated with length of hospital stay and mortality. Former studies have found 4AT > 4 to be a strong predictor of death [35]. We did not find a statistically significant difference in level of care after discharge between patients with and without delirium when correcting for age, sex and NEWS, which could be because delirium was not diagnosed and acknowledged. This finding was not in line with a previous meta-analysis which found delirium to be associated with an increased risk of institutionalisation [6]. A large 2025 study of more than 18,000 hip fracture patients found delirium to be associated with a lower likelihood of returning home within 30 days [44].

### Strengths and limitations

A strength of our study is the high proportion of eligible patients included, as more than 92% of the target population for the study was included, which is high compared to similar studies. Our high inclusion rate is a strength in providing an accurate estimate of delirium prevalence and incidence. Stavanger University Hospital is the only hospital in our catchment area, meaning that hospitalised patients with delirium were not admitted to other hospitals, which could be the case in other countries and cities where several hospitals share a specified catchment area. We have based our formal delirium diagnosis on The Diagnostic and Statistical Manual of Mental Disorders, Fifth Edition´s criteria [1], conducted by two independent geriatricians experienced in delirium diagnosis. This is a strength, because although the sensitivity and specificity of the 4As test is high in previous studies [22], it does not provide a formal delirium diagnosis. For some patients an increased score on the 4As may not represent a delirium, particularly in patients with existing cognitive decline and dementia, where a formal diagnosis of delirium can be challenging [43]. It is well known that delirium estimates differ based on the diagnostic criteria used, with DMS-IV showing higher estimates than DSM-V [4], which was used in our study. It is also a strength that we estimate both a point prevalence and an incidence for delirium, as many studies in recent years provide only a delirium point prevalence [31, 32, 34].

This study also has limitations: it was conducted in a single hospital and only included patients for 5 days and 4 nights, which is a short period, and did not include the weekend. This could have led to a selection bias. We had to balance the need for a high inclusion rate to provide an accurate estimate of prevalence with the secondary aims of examining outcomes for delirium patients. It would be relevant to adjust our outcomes for frailty as well as for comorbidity to explore whether the negative outcomes observed in delirium patients might indeed be caused by delirium itself. To achieve a high inclusion rate, we were given approval from the ethics committee to collect information without obtaining consent from the participants first but were limited to collecting only some predefined data. Our primary aim was to investigate the occurrence of delirium, and we thus chose to prioritise this in the design of our study. It is also a general limitation that the DSM-V do not use operationalised criteria, meaning that for instance the definition of a disturbance in attention and awareness might differ slightly when comparing with other studies. A chart-based delirium diagnosis can also be considered a limitation, as it differs from a bed side interview and clinical examination and does not present firsthand information but presents other clinical personnels descriptions, meaning for instance that we cannot know exactly what informs a description of a patient as confused. Limitations in chart revision for delirium diagnosis have been described previously by Inouyue et al. highlighting that the chart review is particularly challenging in patients with dementia, high delirium risk and critical illness [45]. We have however combined the chart revision with verified screening methods- 4AT, RASS and OSLA, providing a robust method for estimating delirium point prevalence in the ED. No systematic daily delirium screening was conducted after the emergency department, which means that the retrospective diagnosis of delirium in the hospital wards was based on the notes in the medical record. This is a clear limitation as delirium is underreported and we then rely upon on other health care personnel describing characteristics and features of delirium in the health record. Systematically considering delirium daily throughout the hospitalisation could possibly have identified more patients with a hypoactive delirium and thus increased the estimates of delirium incidence.

## Conclusions

We found delirium to be common, with a point prevalence of 14% in the ED and a total occurrence of 21% among hospitalised unselected older adults ≥ 65 years acutely admitted in the study period. Only 8% of those with delirium had a formal delirium diagnosis documented in the discharge summaries. This highlights the need for continuous focus on delirium identification. The 4AT had a quite low sensitivity of 70% in identifying delirium in our study. Patients with delirium showed significantly increased length of stay and higher 9-month mortality. As the population ages, the number of hospital admission and the number of people living with dementia increase, updated estimates of delirium prevalence in unselected cohorts are important to quantify the burden of delirium. Implementation of delirium screening is resource demanding but identifies most patients with delirium and increases the attention and focus on this important condition. Next steps for hospitals that have implemented screening could be validating the screening against a reference standard for delirium diagnosis and further implementation of prevention strategies. Clinicians should be aware that not all patients with delirium present with a positive screening and extra attention should be given to patients presenting with symptoms typical of a delirium and negative screening or those at a particularly high risk of delirium development. Repeated screening or expanded diagnostics could be useful in these situations.

Delirium prevention, identification and management are key aspects of a sustainable health care system facing an increasing proportion of older adults with multifaceted health issues and varying levels of cognitive dysfunction. Future studies should include larger number of patients, diagnose delirium by verified diagnostic criteria and assess as high a percentage of all eligible patients as possible to provide accurate and non-biased estimates of delirium prevalence. Repeated cross-sectional studies could be relevant to examine the changes in delirium prevalence over time.

## Supplementary Information

Supplementary Material 1

Supplementary Material 2

## Data Availability

The dataset analyzed during the current study can upon request to the first author be made available, but only after application to and approval by The Regional Ethical Committee.

## Abbreviations

4AT: The 4 “A” s test
DSM-V: The Diagnostic and Statistical Manual of Mental Disorders, Fifth Edition
ED: Emergency department
IQR: Inter quartile range
NEWS: National Early Warning Score
OSLA: Observational Scale of Level of Arousal
RASS: Richmond Agitation Sedation Scale
SESAM: Centre for Age-Related Medicine
SUH: Stavanger University Hospital
NIH: National Institute of Aging
NICE: National Institute for Health and Care Excellence
SSD: Subsyndromal delirium
