# Supplementary material for: Delirium prevalence and incidence in acutely admitted older patients: an observational cohort study
Source: BMC Geriatr. 2025 Dec 19;26:92. doi: 10.1186/s12877-025-06903-8 (PMC12831450; doi:10.1186/s12877-025-06903-8)
Supplement: Supplementary file 1 — Supplementary Material 1 [file 12877_2025_6903_MOESM1_ESM.docx]

**
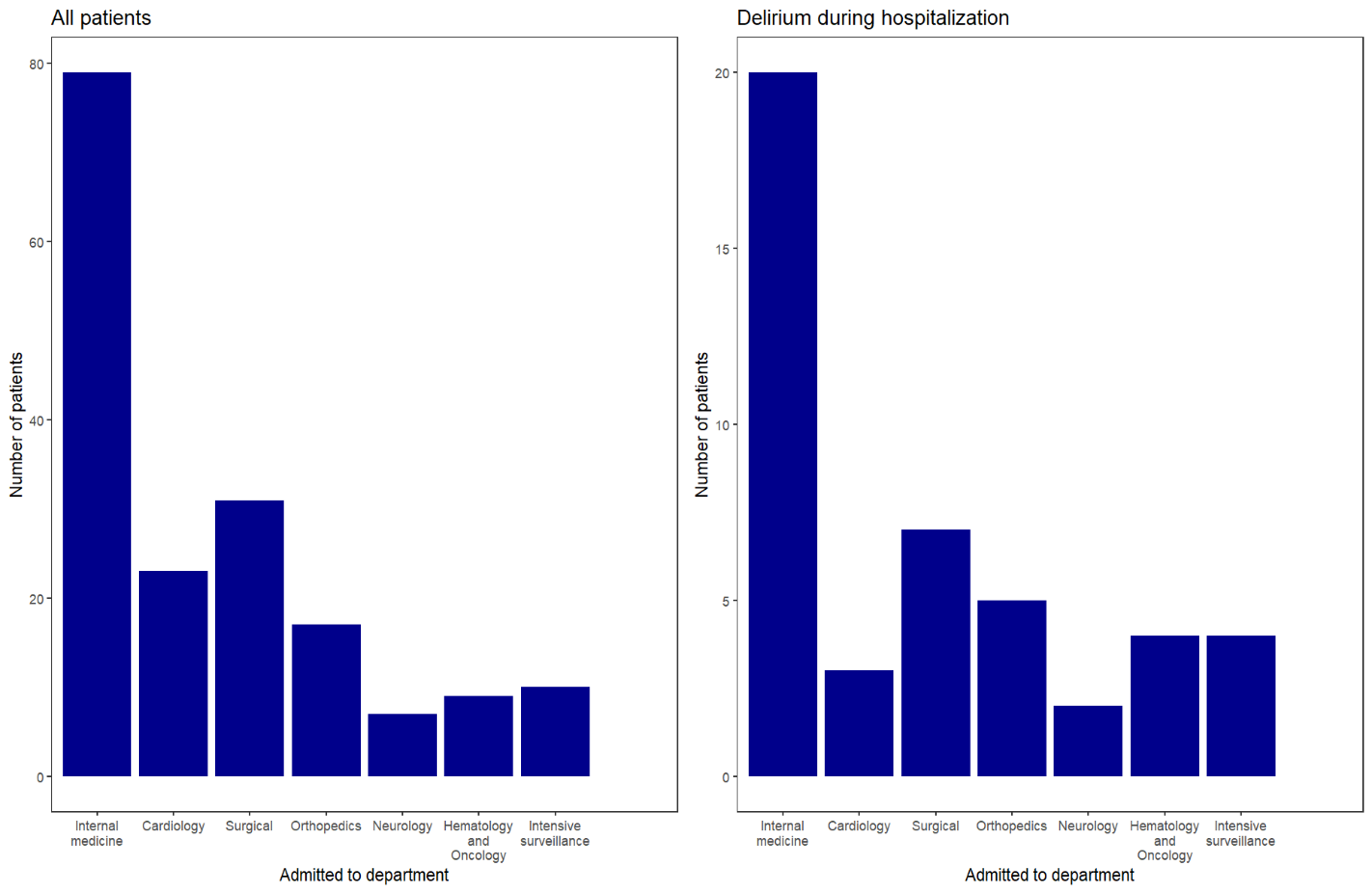
**

**Figure:** Bar chart visualizing which department the patients were admitted to A) for all patients acutely admitted > 65 years in the study period, and B) For the patients acutely admitted > 65 years in the study period with a delirium during the hospitalization.
