# Supplementary material for: Delirium prevalence and incidence in acutely admitted older patients: an observational cohort study
Source: BMC Geriatr. 2025 Dec 19;26:92. doi: 10.1186/s12877-025-06903-8 (PMC12831450; doi:10.1186/s12877-025-06903-8)
Supplement: Supplementary file 2 — Supplementary Material 2 [file 12877_2025_6903_MOESM2_ESM.pdf]

Table 1. Diagnostic algorithm for DSM-V delirium

DSM-V criteria	Test to be performed or information needed	DSM-V criteria fulfilled?	
		YES	NO
A: Disturbance in **attention** (i.e., reduced ability to direct, focus, sustain, and shift attention) and **awareness** (reduced orientation to the environment).	TEST: 4As test- Months of the year in reverse order<br>**Cut off (definition of inattention)**: unable to reach july.<br><br>Observations (documented in medical record):<br>Distractibility. Tendency to lose the tread in conversation. RASS. OSLA.		
B: The disturbance develops over a **short period of time** (usually hours to a few days), represents a **change** from baseline attention and awareness and tends to **fluctuate** in severity during the course of the day.	Information documented in the medical record from patients carers, nurses or doctors;<br>i.e. documented changes in the patients mental state, altered consciousness, sleep-wake cycle disturbances, variability in presentation of these symptoms.<br><br>4As test: "acute change or fluctuating course"?		
C: An additional disturbance in **cognition** (i.e. memory deficit, disorientation, language, visuospatial ability, or perception).	Information from the medical record:<br>Orientation to time, place and person. 4As test: AMT4. **Cut off** $\geq$ 2 mistakes or untestable.<br><br>Any documented episodes of hallucinations/illusions, perceptual disturbances, sleep-wake cycle disturbances, memory disturbances, psychotic symptoms or psychomotor abnormalities.		

D: The disturbances in criteria A and C are **not explained by another pre-existing, established or evolving neurodegenerative disorder** and do not occur in the context of a severely reduced level of arousal, such as coma.	Information from history/medical record.		
E: There is evidence from the history, physical examination, or laboratory findings that the disturbance is a direct **physiologic consequence** of another medical condition, substance intoxication or withdrawal (i.e. because a drug of abuse or to a medication), or exposure to a toxin or is because of multiple aetiologies.	Information from history/medical record.		
Conclusion: **Delirium** based on the tests and information above?	All DSM-V criteria fulfilled	YES	NO
**Subsyndromal delirium** based on the tests and information above?	DSM-V criteria not fulfilled, but evidence of change, in addition to any one of these: a) altered arousal, b) attentional deficits, c) other cognitive change, d) delusions or hallucinations.	YES	NO
